# Cardiac magnetic resonance shows increased adverse ventricular remodeling in younger patients after ST-segment elevation myocardial infarction

**DOI:** 10.1007/s00330-023-09406-5

**Published:** 2023-01-26

**Authors:** Ruifeng Guo, Xiao Wang, Qian Guo, Yan Yan, Wei Gong, Wen Zheng, Guanqi Zhao, Hui Wang, Lei Xu, Shaoping Nie

**Affiliations:** 1 Center for Coronary Artery Disease, Division of Cardiology, Beijing Anzhen Hospital, Capital Medical University, 2 Anzhen Road, Chaoyang District, Beijing, 100029 China; 2 Department of Radiology, Beijing Anzhen Hospital, Capital Medical University, 2 Anzhen Road, Chaoyang District, Beijing, 100029 China

**Keywords:** Ventricular remodeling, Myocardial infarction, Age, Magnetic resonance imaging

## Abstract

**Objectives:**

Young patients account for about half of ST-segment elevation myocardial infarction (STEMI) patients and display a unique risk profile compared with old patients. Whether these differences are related to disparities in ventricular remodeling remains unknown. This study aimed to evaluate age-related differences in ventricular remodeling after primary percutaneous coronary intervention (PPCI) for STEMI.

**Methods:**

In this observational study, consecutive STEMI patients between October 2019 and March 2021 who underwent serial cardiovascular magnetic resonance at index admission (3 to 7 days) and 3 months after PPCI were enrolled. Adverse remodeling was defined as ≥ 10% enlargement in left ventricular end-diastolic volume index (LVEDVi), while reverse remodeling was defined as a decrease in left ventricular end-systolic volume index (LVESVi) of more than 10%.

**Results:**

A total of 123 patients were included and grouped into young (< 60 years, *n* = 71) and old (≥ 60 years, *n* = 52) patients. Despite generally similar baseline structural and infarct characteristics, LVESVi significantly decreased only in old patients during follow-up (*p* = 0.034). The incidence of adverse remodeling was higher (49.3% vs 30.8%, *p* = 0.039), while the incidence of reverse remodeling was lower (31.0% vs 53.8%, *p* = 0.011) in young compared with old patients. Younger age (< 60 years) was associated with a significantly higher risk of adverse remodeling (adjusted OR 3.51, 95% CI 1.41–8.74, *p* = 0.007) and lower incidence of reverse remodeling (adjusted OR 0.42, 95% CI 0.18–0.97, *p* = 0.046).

**Conclusions:**

In STEMI patients undergoing PPCI, young patients are at a higher risk of adverse remodeling and less probably develop reverse remodeling than old patients. Equal or more attention should be paid to young patients with STEMI compared with their older counterparts.

**Key Points:**

• *In STEMI patients undergoing PPCI, young patients displayed unfavorable remodeling compared with old patients*.

• *Young patients are at a higher risk of adverse remodeling and less probably develop reverse remodeling than old patients*.

• *Equal or more attention should be paid to young patients compared with their older counterparts*.

**Supplementary Information:**

The online version contains supplementary material available at 10.1007/s00330-023-09406-5.

## Introduction

Young individuals account for about half of all ST-segment elevation myocardial infarction (STEMI) patients and this proportion is increasing [1]. Although the incidence of acute myocardial infarction has decreased in older populations, there are no similar declines in younger individuals [2]. This group has a unique risk profile with fewer traditional cardiovascular risk factors compared with older populations. Despite timely reperfusion along with optimal medical therapy, STEMI often leads to left ventricular (LV) systolic and/or diastolic dysfunction, followed by ventricular remodeling, which has become one of the main precursors of heart failure [3]. Advanced age is an independent predictor of heart failure and mortality after primary percutaneous coronary intervention (PPCI) for STEMI [4]. However, myocardial infarction conferred a greater relative risk of heart failure in younger compared with older patients [5].

Previous studies have revealed significant differences in cardiovascular risk profiles, the extent of coronary artery disease, pathophysiology of coronary artery occlusion, and clinical outcome in patients with STEMI between age groups [4, 6–12]. Yet, whether these differences are related to disparities in ventricular remodeling, which are known to influence prognosis, remains undetermined [13, 14]. Therefore, we sought to compare serial cardiac magnetic resonance (CMR) data of young (< 60 years) and old (≥ 60 years) patients with STEMI undergoing PPCI to investigate age differences in ventricular remodeling.

## Methods

### Study population

Patients undergoing PPCI for STEMI admitted to Beijing Anzhen Hospital, Capital Medical University were prospectively considered for enrollment between 17 October 2019 and 18 March 2021. STEMI was defined according to the current European Society of Cardiology (ESC) guidelines [15]. CMR scanning was performed at index admission (3 to 7 days) and repeated at 3 months after PPCI. The main CMR exclusion criteria included contraindications to CMR, unstable clinical condition, claustrophobia, patient refusal, and reinfarction or death at any time before follow-up CMR. Patients with incomplete CMR studies or poor image quality were also excluded. Patients were stratified by a cut-off of 60 years referring to the young and the old population according to World Health Organization (WHO) [16]. This study was approved by the ethical committee of Beijing Anzhen Hospital, Capital Medical University, and all subjects provided written informed consent.

### Cardiac magnetic resonance acquisition

Index admission and follow-up CMR were conducted on a 3.0-Tesla electrocardiographically gated CMR imaging system (Ingenia CX, Philips Healthcare, or MR750W, General Electric Healthcare). The imaging protocol consisted of cine images, black blood fat-suppressed T2-weighted (T2w), and late-enhanced images (LGE) 10 min after gadolinium injection (0.2 mmol/kg; Magnevist, Bayer HealthCare Pharmaceuticals Inc. This gadolinium-based contrast agent is still in use in China) at short-axis and long axis views. All sequences were acquired in breath-hold, with a field of view of 350 × 350 mm^2^. The whole left and right ventricle from the mitral annulus to the apex are contained in continuous short-axis slices (8-mm slice thickness, without a gap), with 25–30 phases per cardiac cycle. Long axis planes (2-, 4-, and 3-chamber views) with 5-mm slice thickness had no spacing intersection gap.

Cine CMR was performed using steady-state free precession (SSFP) with repetition time/echo time (TR/TE), 3.2/1.6 ms; flip angle (FA) = 45°; voxel size, 2.0 × 1.6 × 8 mm^3^, and acceleration factor = 1.5 or 3 depending on the number of slices acquired in each breath-hold. Black blood T2w imaging was performed using multishot turbo spin echo (TSE) sequence with TR = 2 heartbeat periods, TE = 75 ms, voxel size = 1.7 × 1.7 × 8 mm^3^, FA = 90°, and acceleration factor = 2. The inversion time of LGE images was optimized to null normal myocardium, and the imaging parameters were as follows: repetition time/echo time, 4.1/1.6 ms; flip angle, 20°; image matrix, 256 × 130.

### Cardiac magnetic resonance analyses

Evaluation of images was carried out using commercially available post-processing software (CVI42, version 5.13, Circle Cardiovascular Imaging Inc.) by observers with at least 3 years of CMR experience (R.G. and Q.G.) who were blinded to all of other details and reviewed by experienced CMR cardiologists (H.W.).

Cine imaging assessed LV function, structure (myocardial mass, left ventricular end-diastolic volume index [LVEDVi], and left ventricular end-systolic volume index [LVESVi] which were calculated as LVEDV or LVESV divided by body surface area, and LV ejection fraction [LVEF]) as well as LV strain, including global longitudinal strain (GLS), global radial strain (GRS), and global circumferential strain (GCS) derived from short-axis and 1–3 slices long-axis images (2-chamber, 3-chamber, and 4-chamber planes). The global strains were calculated as the mean of the respective 16-segment peak values on the software. T2w-STIR was performed to quantify edema (area at risk, AAR) within the territory of the culprit vessel (signal intensity > 2 SDs above the mean signal in remote skeletal muscle) and intramyocardial hemorrhage (IMH) recognized as hypo-enhanced area within the edema. Infarct size is determined as hyper-enhanced myocardium with a signal intensity > 5 SDs of remote normal myocardium. Microvascular obstruction (MVO) is defined as any hypo-enhanced area within an infraction. The extent of the infarction size, IMH, and MVO were quantified as a percentage of LV myocardial mass (%LV). Consequently, the myocardial salvage index (MSI) is calculated as the percentage of the AAR that is not infarcted on LGE images: (AAR − infarct size/AAR) × 100% [17].

Percentage change (%Δ) in LVEDVi and LVESVi was calculated as the difference between the follow-up parameters and the corresponding baseline parameters and expressed as a percentage of the baseline parameters. On the basis of recent literature, after STEMI revascularization, a %ΔLVEDVi value of ≥ 10% showed a strong correlation with clinical outcomes, suggesting this criterion as the preferred CMR-based definition for post-STEMI LV adverse remodeling [13]. On the contrary, reverse remodeling was defined as a decrease in LVESVi of more than 10% on CMR images following revascularized STEMI over a period of time [14].

### Statistical analyses

The distribution of data was scrutinized using the Kolmogorov–Smirnov test and visual methods such as Q-Q plots. For normally distributed continuous variables, data are presented as mean ± SD and compared by the Student’s t-test. Skewed data are expressed as medians with interquartile range (IQR) and compared using the Wilcoxon rank sum test. Categorical variables are presented as the number of cases with corresponding percentages and compared using the chi-square test or Fisher’s exact test. Differences in the changes in CMR parameters between baseline and follow-up in groups were tested by analysis of covariance and Wilcoxon rank sum test. Adverse (≥ 10% increase in LVEDVi at follow-up) or reverse remodeling (≥ 10% decrease in LVESVi at follow-up) were compared between younger and older groups and among age quantiles by chi-square test and Mantel–Haenszel test for linear trend. To evaluate predictors of adverse or reverse remodeling, multiple logistic regression procedures were tested and adjusted by significant baseline characteristics, angiographic, and baseline CMR variables (sex, current smoking, diabetes, anterior infarct, TIMI flow 0–1 pre-PCI, LVEF, and infarct size). Odds ratios (OR) with 95% confidence intervals (CI) were computed. Then, subgroup analyses stratified by clinical status and known anti-remodeling medications were done between dichotomized age (< 60 years vs. ≥ 60 years) and reverse remodeling. A 2-sided *p* value of < 0.05 was deemed significant. SPSS version 26.0. (SPSS Inc) was used for all statistical analyses.

## Results

### Clinical and angiographic characteristics

**Fig. 1:**
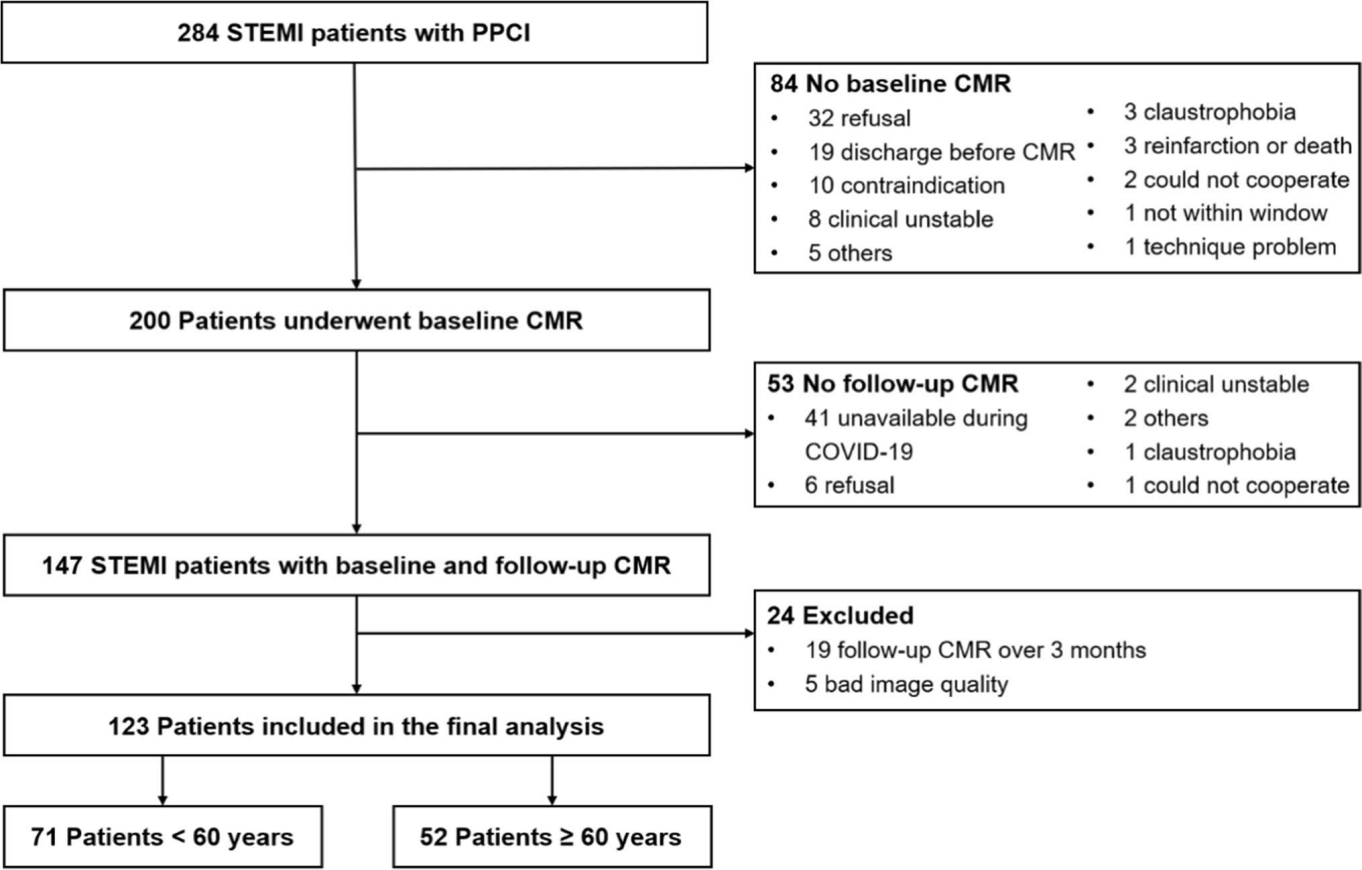
Study flow diagram of selection of eligible subjects. Abbreviations: CMR, cardiac magnetic resonance; PPCI, primary percutaneous coronary intervention; STEMI, ST-elevation myocardial infarction

Of 284 STEMI patients with PPCI screened, 84 patients without baseline CMR and 53 patients without follow-up CMR were excluded. Patients with poor image quality and follow-up CMR > 3 months were also excluded. Finally, a total of 123 patients (median age 57.3 years, 81% men) with serial CMR were included, in which 71 patients were young (< 60 years), and 52 patients were older (≥ 60 years) (Fig. 1). Compared to the old group, young patients were more likely to be men (90.1%) and smoker (63.4%), had higher body mass index, systolic blood pressure, and heart rate and lower peak brain natriuretic peptide levels. The prevalence of traditional risk factors including hypertension and diabetes were numerally lower in young compared with old patients. In general, there were few differences in procedures or medications at discharge (Table 1).

**Table 1:** Baseline characteristics

Parameters	All (*n* = 123)	< 60 years (*n* = 71)	≥ 60 years (*n* = 52)	*p* value
Demographics				
Age, mean (SD), year	57.1 (11.1)	49.5 (7.6)	67.5 (4.8)	< 0.001
Male	100 (81.3)	64 (90.1)	36 (69.2)	0.003
BMI, mean (SD), kg/m.^2^	25.9 (3.2)	26.4 (3.2)	25.1 (3.2)	0.026
Current smoker	62 (50.4)	45 (63.4)	17 (32.7)	0.002
SBP, mean (SD), mmHg	123.1 (14.8)	124.2 (15.7)	121.6 (13.6)	0.35
DBP, mean (SD), mmHg	77.6 (10.2)	79.5 (10.6)	74.9 (9.2)	0.015
HR, mean (SD), beats/min	80.4 (12.6)	84.1 (11.8)	75.3 (12.0)	< 0.001
Medical history				
Hypertension	75 (61)	40 (56.3)	35 (67.3)	0.22
Diabetes	34 (27.6)	16 (22.5)	18 (34.6)	0.14
Dyslipidemia	25 (20.3)	17 (23.9)	8 (15.4)	0.24
Prior stroke	8 (6.5)	2 (2.8)	6 (11.5)	0.053
Prior myocardial infarction	5 (4.1)	3 (4.2)	2 (3.8)	1.00
Previous PCI or CABG	7 (5.7)	3 (4.2)	4 (7.7)	0.46
Clinical presentation				
Anterior infarction	81 (65.9)	49 (69.0)	32 (61.5)	0.39
Killip class				0.64
I	78 (63.4)	48 (61.5)	30 (57.7)	
II	40 (32.5)	21 (29.6)	19 (36.5)	
III	2 (1.6)	1 (1.4)	1 (1.9)	
IV	3 (2.4)	1 (1.4)	2 (3.8)	
Door-to-wire time, median (IQR), min	99.5 (84, 134.25)	93 (81, 122)	105 (88.5, 154)	0.08
Total ischemic time, median (IQR), min	305 (220, 515)	288 (205, 515)	349 (233.5, 521.8)	0.30
Procedures				
Number of diseased arteries				0.42
1	44 (35.8)	25 (35.2)	19 (36.5)	
2	38 (30.9)	25 (35.2)	13 (25)	
3	41 (33.3)	21 (29.6)	20 (38.5)	
Location of culprit lesion				0.34
LAD	81 (65.9)	48 (67.6)	33 (63.5)	
RCA	30 (24.4)	16 (22.5)	14 (26.9)	
LCX	9 (7.3)	4 (5.6)	5 (9.6)	
Reperfusion therapy				
Aspiration thrombectomy	57 (46.3)	30 (42.3)	27 (51.9)	0.29
Balloon angioplasty only	6 (4.9)	2 (7.7)	4 (2.8)	0.41
PCI with stenting	114 (92.7)	66 (93)	48 (92.3)	0.89
TIMI flow grade 0/1 pre-PCI	83 (67.5)	46 (64.8)	37 (71.2)	0.46
TIMI flow grade 3 post-PCI	119 (96.7)	69 (97.2)	50 (96.2)	1.00
GP IIb/IIIa inhibitor	37 (30.1)	17 (23.9)	20 (38.5)	0.08
Medications at discharge				
β blockers	98 (79.7)	58 (81.7)	40 (76.9)	0.52
ACEI or ARB	51 (41.5)	28 (39.4)	23 (44.2)	0.59
ARNI	48 (39)	26 (36.6)	22 (42.3)	0.52
MRA	22 (17.9)	14 (19.7)	8 (15.4)	0.54
Triad therapy	21 (17.1)	14 (19.7)	7 (13.5)	0.36
Preserved EF (≥ 50%)	2 (3.4)	2 (5.1)	0 (0)	0.30
Reduced or mild reduced EF (< 50%)	19 (29.7)	12 (37.5)	7 (21.9)	0.17
Diuretic agent	14 (11.4)	9 (12.7)	5 (9.6)	0.60
SGLT2i	21 (17.2)	12 (17.1)	9 (17.3)	0.98
Statins	122 (99.2)	70 (98.6)	52 (100)	1.00
PCSK9i	12 (9.8)	5 (7)	7 (13.5)	0.24

Triad therapy includes β blockers, ACEI/ARB/ARNI, and MRA

Abbreviations: *ACEI*, angiotensin-converting enzyme inhibitor; *ARB*, angiotensin receptor blocker; *ARNI*, angiotensin receptor/neprilysin inhibitor; *BMI*, body-mass index; *BNP*, brain natriuretic peptide; *DBP*, diastolic blood pressure; *EF*, ejection fraction; *HR*, heart rate; *SBP*, systolic blood pressure; *IQR*, inter-quartile range; *LAD*, left anterior descending; *LCX*, left circumflex artery; *MRA*, mineralocorticoid receptor antagonist; *PCI*, percutaneous coronary intervention; *PCSK9i*, proprotein convertase subtilisin/kexin type 9 inhibitors; *RCA*, right coronary artery; *SD*, standard deviation; *SGLT2i*, sodium-glucose cotransporter 2 inhibitors; *TIMI*, thrombolysis in myocardial infarction

### Index and follow-up CMR findings

The median time from admission and first CMR and follow-up CMR were 4 days and 3.5 months without significant differences between age groups. The LVEF (51.1% vs 46.5%, *p* = 0.03) was higher and GCS (− 13.6% vs − 12.1%, *p*= 0.023) was greater at baseline in young patients than in the old group, whereas LVEF and GCS were similar between groups at follow-up. LVEDVi, LVESVi, LV mass index, infarct size, AAR, MSI, and prevalence or extent of MVO or IMH at baseline and follow-up were similar in both groups (Supplemental Table S1).

**Fig. 2:**
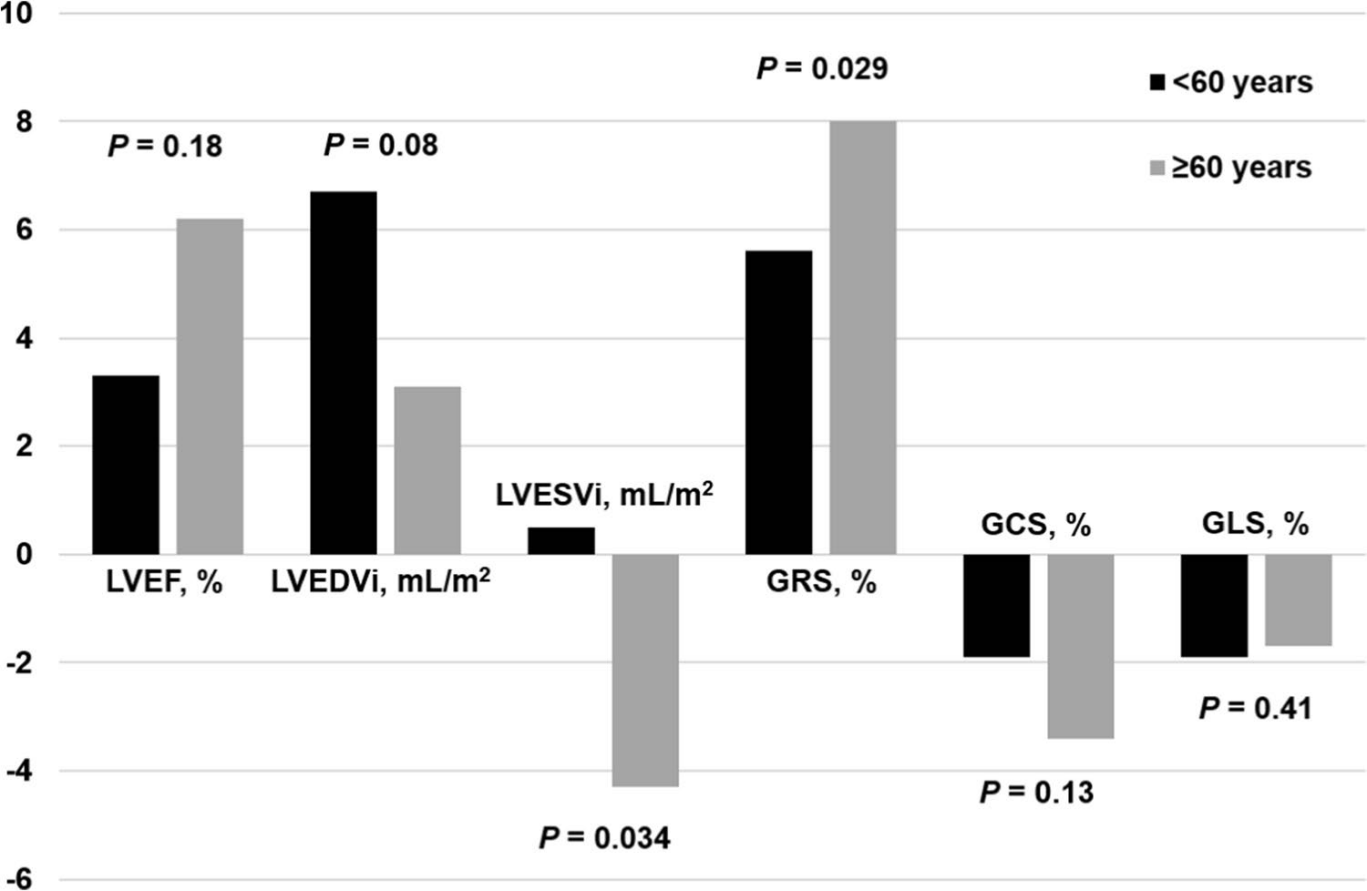
Change in left ventricular structure and function by age at follow-up. Abbreviations: GCS, Global circumferential strain; GLS, Global longitudinal strain; GRS, Global radial strain; LVEDVi, Left ventricular end-diastolic volume index; LVEF, Left ventricular ejection, fraction; LVESVi, Left ventricular end-systolic volume index. Differences between groups were tested with analysis of covariance (ANCOVA)

**Fig. 3:**
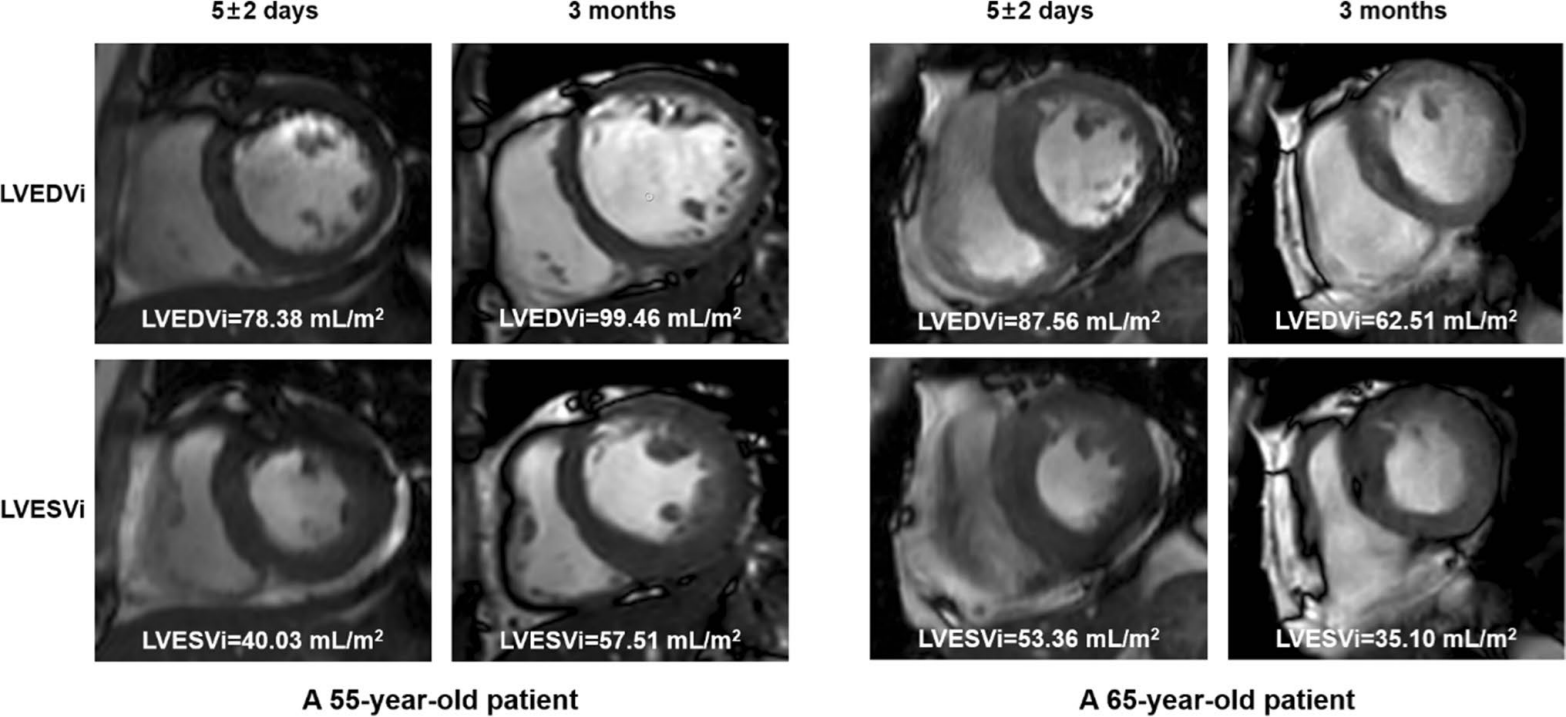
Representative CMR images of young and old patients with typical changes of LVESVi and LVEDVi. Short-axis cine CMR obtained 5 ± 2 days (baseline) and 3 months (follow-up) after STEMI show the course of reverse and adverse remodeling according to age groups. Characteristically, both LVEDVi and LVESVi increased in this younger patient, indicating adverse remodeling and no reverse remodeling. In contrast, both LVEDVi and LVESVi decreased in this older patient, indicating reverse remodeling and no adverse remodeling. The exact size of volume has been shown at the bottom of each graph. Abbreviations: LVEDVi, Left ventricular end-diastolic volume index; LVESVi, left ventricular end-systolic volume index

Changes in CMR parameters were shown in Table 2 and Figs. 2 and 3. At follow-up, ΔLVEDVi had increased numerally more in young patients than in old patients. Meanwhile, LVESVi significantly decreased only in old patients. Correspondingly, there was an absolute increase in LVESVi of 0.5 (− 5.8, 4.7) mL/m^2^ in young patients compared with an absolute decrease of 4.3 (− 9.2, 4.6) mL/m^2^ in old patients (*p* = 0.034). GRS was also significantly improved among old patients (8.0% IQR, 0.3–16.8) compared with young patients (5.6% IQR, 2.3–10.2) (*p* = 0.029). There was no significant difference between groups in the change in LVEF, LV mass index, and change in infarct characteristics.

**Table 2:** Change in CMR parameters between baseline and follow-up

	Median (IQR)		*p* value		
Parameters	All (*n* = 123)	< 60 years (*n* = 71)	≥ 60 years (*n* = 52)	ANCOVA	Wilcoxon rank sum test
Δ LVEF, %	4.5 (− 1.0, 10.7)	3.3 (− 2.0, 8.5)	6.2 (− 0.1, 14.6)	0.18	0.054
Δ LVEDVi, mL/m^2^	5.3 (− 2.9, 12.4)	6.7 (− 1.9, 13.2)	3.1 (− 4.4, 10.1)	0.08	0.12
Δ LVESVi, mL/m^2^	− 0.8 (− 7.3, 4.7)	0.5 (− 5.8, 4.7)	− 4.3 (− 9.2, 4.6)	0.034	0.03
Δ LV mass index, g/m^2^	− 7.4 (− 11.1, − 1.6)	− 7.8 (− 12.1, − 2.5)	− 6.4 (− 9.9, 0.1)	0.10	0.09
Δ GRS, %	6.0 (1.6, 11.8)	5.6 (2.3, 10.2)	8.0 (0.3, 16.8)	0.029	0.11
Δ GCS, %	− 2.5 (− 4.1, − 0.9)	− 1.9 (− 3.7, − 0.2)	− 3.4 (− 5.1, − 1.1)	0.13	0.02
Δ GLS, %	− 1.7 (− 3.3, 0.2)	− 1.9 (− 3.3, − 0.1)	− 1.7 (− 3.5, − 0.8)	0.41	0.46
Δ AAR, % LV mass	− 27.8 (− 37.1, − 17.8)	− 24.8 (− 37.1, − 17.3)	− 27.0 (− 37.4, − 17.8)	0.20	0.84
Δ Infarct size, % LV mass	− 8.5 (− 14.0, − 3.3)	− 8.1 (− 13.3, − 3.6)	− 9.7 (− 16.6, − 2.2)	0.48	0.54
Δ MVO, % LV mass	− 0.02 (− 4.9, 0)	0 (− 3.7, 0)	− 0.2 (− 3.3, 0)	0.72	0.43
Δ IMH, % LV mass	0 (− 0.5, 0)	0 (− 0.33, 0)	0 (0, 0)	0.13	0.22

Abbreviations: *AAR*, area at risk; *GCS*, global circumferential strain; *GLS*, global longitudinal strain; *GRS*, global radial strain; *LVEDV*, left ventricular end-diastolic volume; *LVEDVi*, left ventricular end-diastolic volume index; *LVESV*, left ventricular end-systolic volume; *LVEF*, left ventricular ejection fraction; *LVESVi*, left ventricular end-systolic volume index; *MSI*, myocardial salvage index; *MVO*, microvascular obstruction; *IMH*, intra-myocardial hemorrhage

*ANCOVA*, analysis of covariance

### Association between age and LV adverse and reverse remodeling

**Fig. 4:**
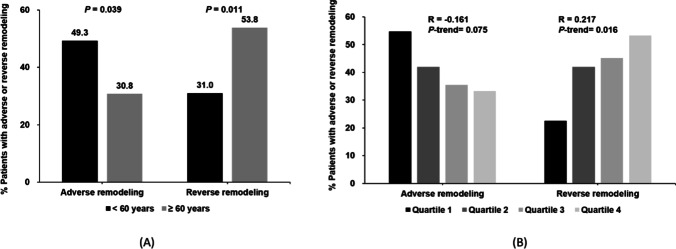
**A**, **B** Frequency of adverse remodeling and reverse remodeling in different age groups

Adverse remodeling occurred in 35 (49.3%) young patients and 16 (30.8%) old patients (*p* = 0.039), while reverse remodeling occurred in 22 (31.0%) young patients and 28 (53.8%) old patients (*p* = 0.011) (Fig. 4A). Multivariable logistic regression analyses revealed that younger age (< 60 years) was independently predictive of adverse remodeling (adjusted OR 3.51, 95% CI 1.41–8.74, *p* = 0.007) and related to lower incidence of reverse remodeling (adjusted OR 0.42, 95% CI 0.18–0.97, *p* = 0.046) (Table 3). Furthermore, represented as a continuous variable, age remained significantly associated with a lower risk of adverse remodeling (adjusted OR for 1-year increase = 0.95, 95% CI 0.91–0.99, *p* = 0.017) and higher incidence of reverse remodeling (adjusted OR for 1-year increase = 1.05, 95% CI 1.00–1.09, *p* = 0.039) (Table 3).

**Table 3:** Multivariate logistic regression analyses for adverse and reverse remodeling

Age as continuous variable (per year)					Age as dichotomous variable (< 60 years vs. ≥ 60 years)			
Variables	Adverse remodeling		Reverse remodeling		Adverse remodeling		Reverse remodeling	
	OR (95% CI)	*p* value	OR (95% CI)	*p* value	OR (95% CI)	*p* value	OR (95% CI)	*p* value
Age	0.95 (0.91, 0.99)	0.017	1.05 (1.00, 1.09)	0.039	3.51 (1.41, 8.74)	0.007	0.42 (0.18, 0.97)	0.046
Male	0.52 (0.16, 1.64)	0.27	0.56 (0.18, 1.78)	0.33	1.69 (0.55, 5.20)	0.36	0.47 (0.15, 0.14)	0.19
Diabetes	0.54 (0.21, 1.39)	0.20	0.98 (0.40, 2.38)	0.96	1.96 (0.56, 5.07)	0.17	1.03 (0.42, 2.53)	0.95
Current smoking	0.71 (0.29, 1.71)	0.71	1.45 (0.59, 3.54)	0.42	1.48 (0.60, 3.65)	0.39	1.42 (0.58, 3l46)	0.44
Anterior infarct	1.32 (0.55, 3.18)	0.54	1.32 (0.54, 3.23)	0.54	1.43 (0.59, 3.45)	0.43	1.16 (0.48, 2.80)	0.73
TIMI flow 0–1 pre-PCI	0.93 (0.39, 2.21)	0.86	0.98 (0.41, 2.35)	0.97	0.92 (0.38,1.07)	0.86	0.98 (0.41, 2.33)	0.96
LVEF, %	0.97 (0.93, 1.01)	0.13	0.93 (0.88, 0.98)	0.006	0.97 (0.92, 1.01)	0.12	0.93 (0.89, 0.98)	0.007
Infarct size, % of LV	1.03 (0.99, 1.07)	0.12	0.95 (0.91, 0.99)	0.010	1.03 (0.99, 1.07)	0.15	0.95 (0.91, 0.99)	0.016

Abbreviations: *CI*, confidence interval; *LVEF*, left ventricular ejection fraction; *OR*, odds ratio

In addition, the incidence of adverse remodeling tended to decrease with the rising quartile levels of age (54.8%, 41.9%, 35.5%, 33.3%, *p* for trend = 0.075), and the rate of reverse remodeling significantly increased with the rising quartile levels of age (22.6%, 41.9%, 45.2%, 53.3%, *p* for trend = 0.016) (Fig. 4B). After multivariable adjustment, the probability of reverse remodeling was lower in the youngest patients (quartile 1 compared with quartile 4, adjusted OR 0.26, 95% CI 0.08–0.77, *p* = 0.016) (Supplemental Table S2).

In subgroup analyses stratified by baseline characteristics and known anti-remodeling medication, younger age correlated with a lower incidence of reverse remodeling in men (OR 0.42, 95% CI 0.18–0.98, *p* = 0.046), patients with reduced LVEF (< 50%) (OR 0.36, 95% CI 0.13–0.99, *p* = 0.048) or dyslipidemia (OR 0.14, 95% CI 0.02–0.94, *p* = 0.043). Interestingly, when triple anti-remodeling drugs (β blockers, ACEI/ARB/ARNI, and mineralocorticoid receptor antagonist [MRA]) (OR 0.40, 95% CI 0.18–0.91, *p* = 0.028) or sodium-glucose cotransporter 2 inhibitors (SGLT2i) (OR 0.36, 95% CI 0.16–0.81, *p* = 0.014) was not administered, young patients showed less reverse remodeling. This was not evident in patients taking those drugs (Supplemental Fig. 1).

## Discussion

In the present study, we used serial CMR data to show the impact of age on ventricular remodeling in STEMI with PPCI. Although baseline structural parameters were similar between young and old patients, young patients displayed less improvement in LVESVi and GRS compared with old patients at follow-up. Adverse remodeling was more common in the young group, while reverse remodeling was more frequent in the old group, a similar trend was also observed when subdividing age into quantiles. Younger age was significantly associated with a higher risk of adverse remodeling and a lower incidence of reverse remodeling. Specifically, the use of anti-remodeling drugs might promote reverse remodeling in younger patients compared with older patients.

Myocardial infarction remains one of the most common causes of adverse outcomes in young individuals [2]. The way in which it affects prognosis is a complex multifactorial process known as cardiac remodeling. Two echocardiography studies have reported that adverse remodeling occurred more [6] or equally [11] often in older patients (> 70 years) than younger patients irrespective of the type of reperfusion therapy (thrombolysis or PCI). However, in the contemporary era of PPCI and intensive medical therapy, few studies compared the differences in ventricular remodeling between young and old patients. Using CMR with more accurate myocardial evaluation, there was no substantial difference in baseline infarct size, AAR, MSI and MVO (myocardial injury) between young and older patients with STEMI [4, 18]. Our findings are consistent with those data. Myocardial strain, the assessment of cardiac deformation and function, is more sensitive than other parameters to identify subtle LV dysfunction resulting in adverse remodeling [19]. In the present study, despite similar infarct characteristics (infarct size, MVO, and IMH), improvement of GRS in younger patients was significantly less than old patients, which might partially explain the unfavorable ventricular remodeling in the younger group compared with the older group.

Cardiac remodeling involves structural, functional, and molecular changes. At the cellular and molecular levels, it is characterized by compensatory hypertrophy of cardiac myocytes and hyperplasia of cardiac fibroblasts. In the rat myocardial infarction model, aged rats showed lower rates of rise and fall in LV pressure, less pronounced expression of atrial natriuretic factor (ANF, a marker of hypertrophy), and preserved heart function depending on remodeling, showing that aged rats well compensate for the hemodynamic overload induced by myocardial infarction [20]. Meanwhile, Gould et al [21] reported that infarct expansion and septal hypertrophy following myocardial infarction seen in the older mice, but not in the younger counterparts, could be improved with pharmacologic therapy (ACEI).

It should be noted that the proportion of females is lower in young patients in this study. Women have a lower propensity to develop spherical geometry and left ventricular dysfunction, and remodeling seems less pronounced in women. Estrogens could play a protective role in ventricular remodeling even after menopause because of persisting intramyocardial synthesis in women [22]. Even so, men and women with STEMI show a similar incidence of LV remodeling in a large cohort of 1995 STEMI patients (48% in men and 48% in women) [23]. Thus, although modest, the impact of sex on the association of age with adverse remodeling should be considered. The suggestion of greater adverse remodeling in young patients should be regarded with caution till more evidence appears from women-dominated cohorts. In terms of cardiovascular risk factors, the prevalence of stroke and diabetes in old patients was numerically higher than that of young patients. A prior echocardiography study found that the diabetes group had less adverse remodeling after STEMI compared with the non-diabetes group [24]. Thus, it brings out the hypothesis that coronary collateral circulation in diabetes [25–27]or angiogenesis in prior stroke [28] and hence preconditioning [29] (one of the most effective ways of protecting the heart against ischemia–reperfusion injury) might increase ischemic tolerance during the acute phase of STEMI in older patients resulting in favorable remodeling, while the occlusion of the coronary artery in young patients more likely results from rapid disease progression [2].

Age was an independent predictor of major adverse cardiac events (MACE) even after adjustment for CMR infarct characteristics [4]. Although the absolute risk and incidence for MACE after STEMI is higher in older patients with considerable age-related multimorbidity and mortality, previous myocardial infarction carries both a stronger relative risk and a greater attributable risk for poor prognosis among young people than older people. Notably, a combined population-based cohort study of Framingham Heart Study, PREVEND, and MESA showed that myocardial infarction is responsible for a greater relative risk of heart failure in younger (< 55 years) compared with older participants (hazard ratio 3.30, 95% CI 1.77 to 6.14; *p* < 0.001)^5^. Ventricular remodeling is one of the main precursors of heart failure post-myocardial infarction. In the current study, we found relatively greater LV volume enlargement in younger patients, suggesting that unfavorable cardiac remodeling post-STEMI might play some role in the relatively greater risk of heart failure. We believe this should be the focus of additional research in the future. In fact, the prognosis for younger patients with myocardial infarction is not benign. The out-of-hospital mortality from ischemic heart disease is significantly higher in very young patients than in old patients [30] In-hospital mortality among patients aged 35–49 years was 2.5% and twice as that among patients aged 60–64 years in Poland [31]. Generally, short-term outcomes post-STEMI at a ‘‘young’’ age may be good but longer prognosis is relatively poor. There is an alarming drop in long-term survival among young myocardial infarction patients after a median follow-up of 11.3 years with mortality exceeding 29% and only 19% of subjects with event-free survival at 15 years [32].

The exploratory data of subgroup analyses indicated that young patients showed a lower probability of reverse remodeling in patients without taking triple anti-remodeling drugs (β blockers, ACEI/ARB/ARNI, and MRA), or SGLT2i. Modulation of the renin–angiotensin–aldosterone and sympathetic nervous systems with ACEI/ARB, β blockers, and MRA has been shown to protect against adverse remodeling and improve survival. These drugs and combinations are recommended in guidelines as cornerstone therapies for STEMI [15] and heart failure [33]. ARNI as a replacement for ACEI in suitable patients has also shown the potential effect of reverse remodeling [34]. Additionally, two landmark trials of SGLT2i have shown a reduced risk of cardiovascular death and worsening heart failure in patients with reduced ejection fraction [35, 36]. This was also true for patients with preserved heart failure [37]. Noteworthy, ischemic heart disease accounted for more than half of the cause of heart failure in those trials [35, 36]. Although the SGLT2i trials focusing on STEMI were still ongoing (NCT03591991 and NCT05045274), we speculate the protective effects of ventricular remodeling might explain the results of subgroup analysis, which indicate no differences in reverse remodeling in patients taking SGLT2i. This is hypothesis-generating and opens the field to further research.

### Limitations

We also acknowledge several limitations. First, selection bias is inherent in this retrospective study. The CMR images of the STEMI population were not consecutively included due to insufficient image quality or lack of examination. Second, the analysis was performed in a single center with a limited and male-dominated population. These exploratory results must be confirmed in a large group in the future. Third, the proportion of male patients was different between young and old groups, although multivariable adjustment by including sex as a covariate did not change the association between age and adverse or reverse remodeling. Fourth, we did not possess the images of quantitative T1 mapping as well as T2 or T2 ∗ mapping which had been provided for advanced infarct characterization including cellular and extracellular components with a potential mechanism for ventricular remodeling post-STEMI.

## Conclusions

Among patients with STEMI undergoing PPCI, young patients are at a higher risk of adverse remodeling and less probably develop reverse remodeling than old patients. Equal or more attention should be paid to young patients with STEMI compared with their older counterparts.

## Supplementary Information

Below is the link to the electronic supplementary material.

Supplementary file1 (DOCX 209 KB)

## Data Availability

Data generated or analyzed during the study are available from the corresponding author by request.

## Abbreviations

AAR: Area at risk
ACEI: Angiotensin-converting enzyme inhibitor
ARB: Angiotensin receptor blocker
ARNI: Angiotensin receptor/neprilysin inhibitor
BMI: Body-mass index
BNP: Brain natriuretic peptide
CI: Confidence intervals
CMR: Cardiac magnetic resonance
DBP: Diastolic blood pressure
ESC: European Society of Cardiology
GCS: Global circumferential strain
GLS: Global longitudinal strain
GRS: Global radial strain
HR: Heart rate
IMH: Intra-myocardial hemorrhage
IQR: Inter-quartile range
LAD: Left anterior descending
LCX: Left circumflex artery
LGE: Late-enhanced images
LV: Left ventricular
LVEDVi: Left ventricular end-diastolic volume index
LVEF: Left ventricular ejection fraction
LVESVi: Left ventricular end-systolic volume index
MRA: Mineralocorticoid receptor antagonist
MSI: Myocardial salvage index
MVO: Microvascular obstruction
OR: Odds ratios
PCI: Percutaneous coronary intervention
PCSK9i: Proprotein convertase subtilisin/kexin type 9 inhibitors
PPCI: Primary percutaneous coronary intervention
RCA: Right coronary artery
SBP: Systolic blood pressure
SD: Standard deviation
SGLT2i: Sodium-glucose cotransporter 2 inhibitors
SPSS: Steady-state free precession
STEMI: ST-segment elevation myocardial infarction
TIMI: Thrombolysis in myocardial infarction
TR/TE: Repetition time/echo time
TSE: Turbo spin echo
WHO: World Health Organization

## Declarations

### Guarantor

The scientific guarantor of this publication is Professor Shaoping Nie.

E-mail: spnie@ccmu.edu.cn.

### Statistics and biometry

One of the authors (Dr Wen Zheng) has significant statistical expertise.

### Methodology

• prospective

• observational

• performed at one institution
