# Supplementary material for: Cardiac magnetic resonance shows increased adverse ventricular remodeling in younger patients after ST-segment elevation myocardial infarction
Source: Eur Radiol. 2023 Jan 26;33(7):4637–47. doi: 10.1007/s00330-023-09406-5 (PMC10289996; doi:10.1007/s00330-023-09406-5)
Supplement: Supplementary file 1 — Supplementary file1 (DOCX 209 KB) [file 330_2023_9406_MOESM1_ESM.docx]

# Supplemental material

## Supplemental table S1. CMR results at admission and follow-up

| **Parameters** | **All (n = 123)** | **<60 years (n = 71)** | **≥60 years (n = 52)** | ***P* value** |
| --- | --- | --- | --- | --- |
| **Index CMR findings** |  |  |  |  |
| Intervals, median (IQR), day | 4 (3, 5) | 4 (3, 5) | 4 (3, 5) | 0.79 |
| **LV volume and function** |  |  |  |  |
| LVEF, mean (SD), % | 49.1 (11.5) | 51.1 (10.9) | 46.5 (12.0) | 0.03 |
| LVEDVi, mean (SD), mL/m^2^ | 69.8 (14.0) | 70.0 (12.3) | 69.6 (16.1) | 0.89 |
| LVESVi, mean (SD), mL/m^2^ | 35.7 (11.4) | 34.5 (10.7) | 37.2 (12.2) | 0.19 |
| LV mass index, mean (SD), g/m^2^ | 67.7 (12.7) | 68.9 (10.5) | 66.0 (15.2) | 0.24 |
| GRS, mean (SD), % | 18.5 (8.6) | 18.8 (8.4) | 18.0 (8.9) | 0.60 |
| GCS, mean (SD), % | -12.9 (3.6) | -13.6 (3.3) | -12.1 (3.9) | 0.023 |
| GLS, mean (SD), % | -8.4 (3.1) | -8.5 (3.2) | -8.2 (3.0) | 0.62 |
| **Edema and infarct characteristics** |  |  |  |  |
| AAR, mean (SD), % LV mass | 62.8 (13.1) | 61.4 (12.8) | 64.9 (13.1) | 0.11 |
| Infarct size, mean (SD), % LV mass | 37.3 (13.1) | 35.9 (13.2) | 39.2 (12.9) | 0.16 |
| Presence of MVO, n (%) | 67 (54.5) | 36 (50.7) | 31 (59.6) | 0.33 |
| MVO, median (IQR), % LV mass | 0.26 (0, 3.56) | 0.05 (0, 3.7) | 0.34 (0, 3.30) | 0.71 |
| Presence of IMH, n (%) | 44 (37.3) | 26 (39.4) | 18 (34.6) | 0.59 |
| IMH, median (IQR), % LV mass | 0 (0, 0.69) | 0 (0, 0.70) | 0 (0, 0.48) | 0.60 |
| MSI, median (IQR) | 42.0 (30.6, 50.7) | 42.7 (13.7) | 40.5 (13.1) | 0.39 |
| **Follow-up CMR findings** |  |  |  |  |
| Intervals, median (IQR), day | 104 (90, 127) | 100 (89, 116) | 107 (90.5, 152) | 0.13 |
| **LV volume and function** |  |  |  |  |
| LVEF, mean (SD), % | 54.7 (10.3) | 54.9 (9.8) | 54.4 (11.0) | 0.85 |
| LVEDVi, mean (SD), mL/m^2^ | 73.9 (17.2) | 75.7 (17.1) | 71.4 (17.2) | 0.17 |
| LVESVi, mean (SD), mL/m^2^ | 34.5 (14.3) | 35.2 (15.0) | 33.6 (13.5) | 0.52 |
| LV mass index, mean (SD), g/m^2^ | 61.4 (10.2) | 61.2 (9.5) | 61.7 (11.0) | 0.77 |
| GRS, mean (SD), % | 25.3 (9.1) | 24.2 (7.8) | 26.9 (10.5) | 0.10 |
| GCS, mean (SD), % | -15.5 (3.3) | -15.5 (3.0) | -15.5 (3.6) | 0.92 |
| GLS, mean (SD), % | -9.9 (3.3) | -10.2 (2.7) | -9.6 (3.8) | 0.33 |
| **Edema and infarct characteristics** |  |  |  |  |
| Infarct size, mean (SD), % LV mass | 27.8 (11.5) | 26.5 (11.1) | 29.6 (11.9) | 0.14 |
| Presence of MVO, n (%) | 36 (29.3) | 22 (31.0) | 14 (26.9) | 0.63 |
| MVO, median (IQR), % LV mass | 0 (0, 0.06) | 0 (0, 0.15) | 0 (0, 0.05) | 0.52 |
| Presence of IMH, n (%) | 28 (24.6) | 16 (23.9) | 12 (25.5) | 0.84 |
| IMH, median (IQR), % LV mass | 0 (0, 0.05) | 0 (0, 0) | 0 (0, 0.7) | 0.68 |

Abbreviations: AAR, area at risk; GCS, global circumferential strain; GLS, global longitudinal strain; GRS, global radial strain; LVEDV, left ventricular end diastolic volume; LVEDVi, left ventricular end diastolic volume index; LVESV, left ventricular end systolic volume; LVEF, left ventricular ejection fraction; LVESVi, left ventricular end systolic volume index; MSI, myocardial salvage index; MVO, microvascular obstruction; IMH, intra-myocardial hemorrhage; IQR, inter-quartile range; SD, standard deviation.

## Supplemental table S2. Association between age and ventricular remodeling.

|  | **Adverse remodeling, n (%)** | **Adjusted**  **OR (95% CI)** | ***P* value** | **Reverse remodeling, n (%)** | **Adjusted**  **OR (95% CI)** | ***P* value** |
| --- | --- | --- | --- | --- | --- | --- |
| Quartile 1 | 17 (54.8) | 4.58 (1.26, 16.64) | 0.021 | 7 (22.6) | 0.26 (0.08, 0.77) | 0.016 |
| Quartile 2 | 13 (41.9) | 2.20 (0.66, 7.37) | 0.20 | 13 (41.9) | 0.69 (0.22, 2.23) | 0.54 |
| Quartile 3 | 11 (35.5) | 1.54 (0.47, 5.00) | 0.48 | 14 (45.2) | 0.79 (0.25, 2.49) | 0.69 |
| Quartile 4 | 10 (33.3) | 1.00 (Ref) |  | 16 (53.3) | 1.00 (Ref) |  |
| *P* for trend |  |  | 0.12 |  |  | 0.22 |

Abbreviations: CI, confidence interval; LVEF, left ventricular ejection fraction; OR, odds ratio.

Model was adjusted for sex, current smoking, diabetes, anterior infarct, TIMI flow 0-1 pre-PCI, LVEF, and infarct size (% LV)

## Supplemental Figure 1. Forest plot depicting associations between age and reverse remodeling in patients stratified by baseline characteristics and anti-remodeling medication.


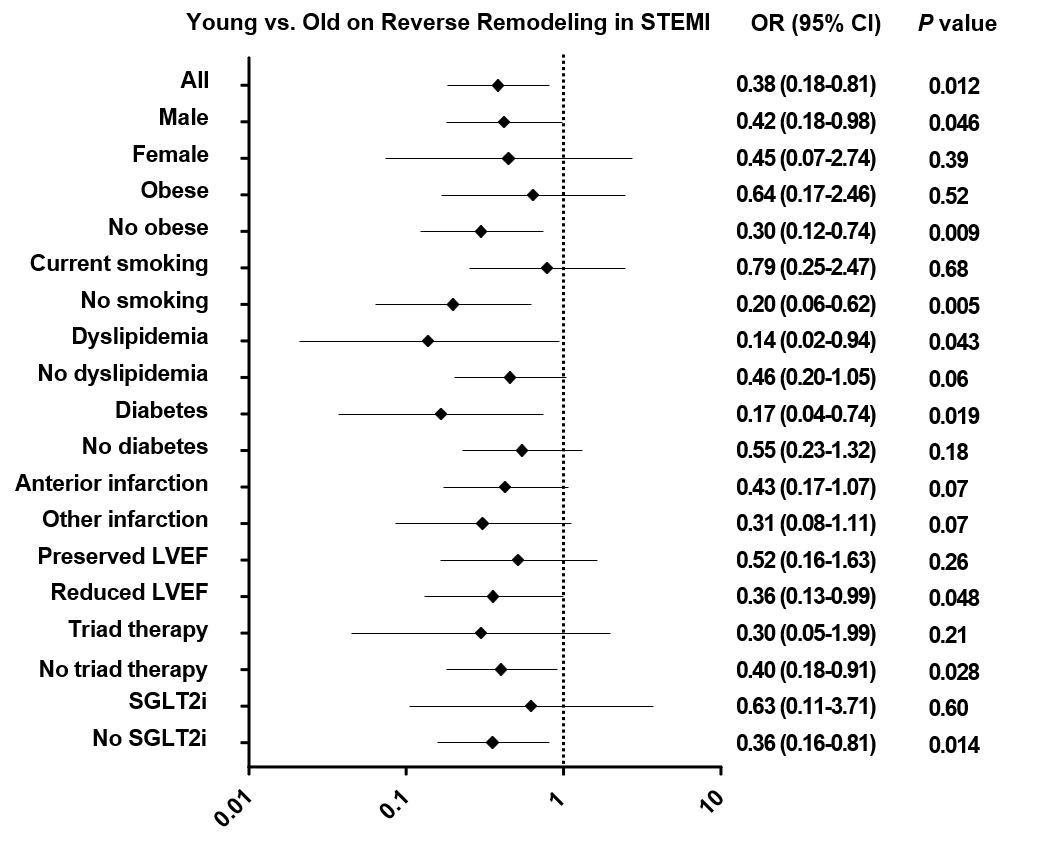


Abbreviations: ACEI, angiotensin converting enzyme inhibitor; ARB, angiotensin receptor blocker; ARNI, angiotensin receptor/neprilysin inhibitor; LVEF, left ventricular ejection fraction; MRA, mineralocorticoid receptor antagonist; SGLT2i, sodium-glucose cotransporter 2 inhibitors.
